# Content validity of preference-based measures for economic evaluation in chronic obstructive pulmonary disease

**DOI:** 10.1186/s12955-021-01744-6

**Published:** 2021-03-20

**Authors:** Ava Mehdipour, Sachi O’Hoski, Marla K. Beauchamp, Joshua Wald, Ayse Kuspinar

**Affiliations:** 1 School of Rehabilitation Science, McMaster University, 1400 Main St. W. Room 435, IAHS, Hamilton, ON L8S 1C7 Canada; 2 Respiratory Research, West Park Healthcare Centre, Toronto, ON M6M 2J5 Canada; 3 Firestone Institute for Respiratory Health, 50 Charlton Ave E, Hamilton, ON L8N 4A6 Canada; 4 Department of Medicine, McMaster University, Hamilton, ON Canada

**Keywords:** COPD, HRQoL, Preference-based measures, Content validity

## Abstract

**Background:**

Generic preference-based measures (GPBMs) are health-related quality of life (HRQoL) measures commonly used to evaluate the cost-utility of interventions in healthcare. However, the degree to which the content of GPBMs reflect the HRQoL of individuals with chronic obstructive pulmonary disease (COPD) has not yet been assessed. The purpose of this study was to examine the content and convergent validity of GPBMs in people with COPD.

**Methods:**

COPD patients were recruited from healthcare centers in Ontario, Canada. The Patient-Generated Index (PGI) (an individualized HRQoL measure) and the RAND-36 (to obtain SF-6D scores; a GPBM) were administered. Life areas nominated with the PGI were coded using the International Classification of Functioning Disability and Health and mapped onto GPBMs.

**Results:**

We included 60 participants with a mean age of 70 and FEV1% predicted of 43. The mean PGI score was 34.55/100 and the top three overarching areas that emerged were: ‘mobility’ (25.93%), ‘recreation and leisure’ (25.19%) and ‘domestic life’ (19.26%). Mapping of the nominated areas revealed that the Quality of Well-Being scale covered the highest number of areas (84.62%), Health Utilities Indices covered the least (15.38% and 30.77%) and other GPBMs covered between 46 and 62%. A correlation of 0.32 was calculated between the SF-6D and the PGI.

**Conclusions:**

The majority of GPBMs covered approximately half of the areas reported as being important to individuals with COPD. When areas relevant to COPD are not captured, HRQoL scores generated by these measures may inaccurately reflect patients’ values and affect cost-effectiveness decisions.

## Background

Health-related quality of life (HRQoL) is “an individual’s perception of how an illness and its treatment affect the physical, mental and social aspects of his or her life” [1]. Different methods of measuring HRQoL have been developed and can be used in research to assign a value to one’s overall HRQoL. Among these methods are generic preference-based measures (GPBMs), which are patient-reported outcome measures of HRQoL that can be used for cost-utility analyses of different interventions [2]. Some well-known GPBMs are the EuroQol 5-Dimensions (EQ-5D), the Six-Dimensional Short Form Survey (SF-6D) and the Health Utilities Index Mark 3 (HUI3) [3]. They are typically anchored from 0.0 (death) to 1.0 (perfect-health), and this value of HRQoL can be used to calculate quality-adjusted life years (QALYs) for an intervention by multiplying it by the number of years the intervention is predicted to extend life. QALYs can be used by healthcare professionals and policymakers to make decisions about resource allocation and implementation of interventions.

Individuals with chronic obstructive pulmonary disease (COPD) experience respiratory symptoms, such as cough, difficulty breathing and fatigue, which have been found to affect HRQoL [4, 5]. Luckily, many treatments have shown to increase health status in people with COPD [6]. The use of GPBMs in COPD can help determine which treatments are more effective in terms of both quality and quantity of life. However, before a measure is used to make cost-effectiveness decisions for a specific population, its psychometric properties should be tested to ensure its reliability and validity [7]. Content validity of GPBMs in people with COPD has not yet been evaluated [8] and is a fundamental step in establishing a measure’s validity as it assesses whether the measure reflects the construct under study [9]. Therefore, the primary objective of this study is to assess the content validity of GPBMs by estimating the extent to which GPBMs capture domains of quality of life that are important to individuals with COPD, as measured by the Patient-Generated Index (PGI).

The SF-36 is a widely used health profile in research studies and clinical trials [10–13]. It is easy to use (can be self-administered and completed within 5 min) and has been translated and adapted in several countries, making it widely available [10, 14]. Preference-based scores can easily be obtained from the SF-36 data [15] and be used to make cost-effectiveness comparisons for different disease groups and populations [16]. Preference-based scores obtained from the SF-36 are known as SF-6D scores [15]. A recent systematic review demonstrated the need for further research on the performance of the SF-6D in COPD [8]. Therefore, the secondary objective of this study is to examine the convergent validity of a well-known GPBM; the SF-6D [3], against the PGI.

## Methods

### Participants

Participants were recruited from outpatient clinics and pulmonary rehabilitation programs at two academic centers in Ontario. Eligibility criteria for the study included: 1) over the age of 18, 2) a clinical physician-diagnosis of COPD, and 3) smoking history of at least 10 pack-years. Individuals who were not able to speak/understand English and those with a severe disability (caused by a musculoskeletal or neurological condition unrelated to their COPD) were excluded.

### Outcome measures

#### Sociodemographic and clinical characteristics

Sociodemographic information, such as sex, age, number of pack years, oxygen use and mobility aid use, and clinical information, such as comorbidities and spirometry results (i.e., forced expiratory volume in one second (FEV1), forced vital capacity (FVC)), were obtained.

#### The Patient-Generated Index (PGI)

The PGI has been utilized in previous content validity studies to identify areas of quality of life important to individuals with chronic conditions [17–19]. This individualized measure of HRQoL was administered in three stages. First, participants were asked to list up to five most important areas of their life affected by their COPD, with the last/sixth item being: ‘all other areas of life that are not mentioned above’. Second, participants were asked to rate each area on a scale from 0 (the worst you could imagine) to 10 (exactly as you would like it to be), relative to the past month. Third, participants were given 12 imaginary points and asked to distribute these points among the areas which they would like to have improved; more points being allocated to areas with more hopes of improvement. The rating of each area and the proportion of complementary points allocated were multiplied and summed to produce a total score of HRQoL on a scale from 0 to 10; with higher scores indicating better HRQoL [20]. This score is typically reported as a percentage [21].

#### The Six-Dimensional Short Form Survey (SF-6D)

The SF-6D is a commonly used GPBM, developed by Brazier et al. [15, 22], from the SF-36 (generic health profile). The SF-6D defines 18,000 health states and items cover 6 dimensions: physical functioning, role limitation, social functioning, pain, mental health and vitality [23, 24]. The RAND-36, a distributable version of the SF-36, was used to obtain SF-6D scores as recommended by the developers [25]. The RAND-36 is a 36-item questionnaire that covers various domains of HRQoL, across 8 scales, varying from physical functioning to mental health and social functioning, summed into 2 subscales (Physical and Mental Health) [26]. Scores obtained from the RAND-36 were transformed to SF-6D scores using an algorithm developed by Kharroubi et al. [27], using non-parametric Bayesian preference weights. The SF-6D produces a HRQoL score from 0.2 (worst possible health state) to 1.0 (perfect health state) [27]. Permission to use the SF-6D algorithm was obtained from the developers.

### Procedure

Eligible participants who provided informed consent completed the PGI and the RAND-36 in person or over the phone. The areas reported from the PGI were coded independently by two reviewers (AM and SO) using the World Health Organization’s International Classification of Functioning, Disability and Health (ICF) [28]. A third reviewer (AK) was consulted if agreement between the reviewers was not reached. The most specific code was selected for each reported area, and if the reported area covered more than one code, then all codes were stated. Similar codes were then pooled together (e.g., ‘recreation and leisure, unspecified’ and ‘recreation and leisure, other specified’).

**Fig. 1:**
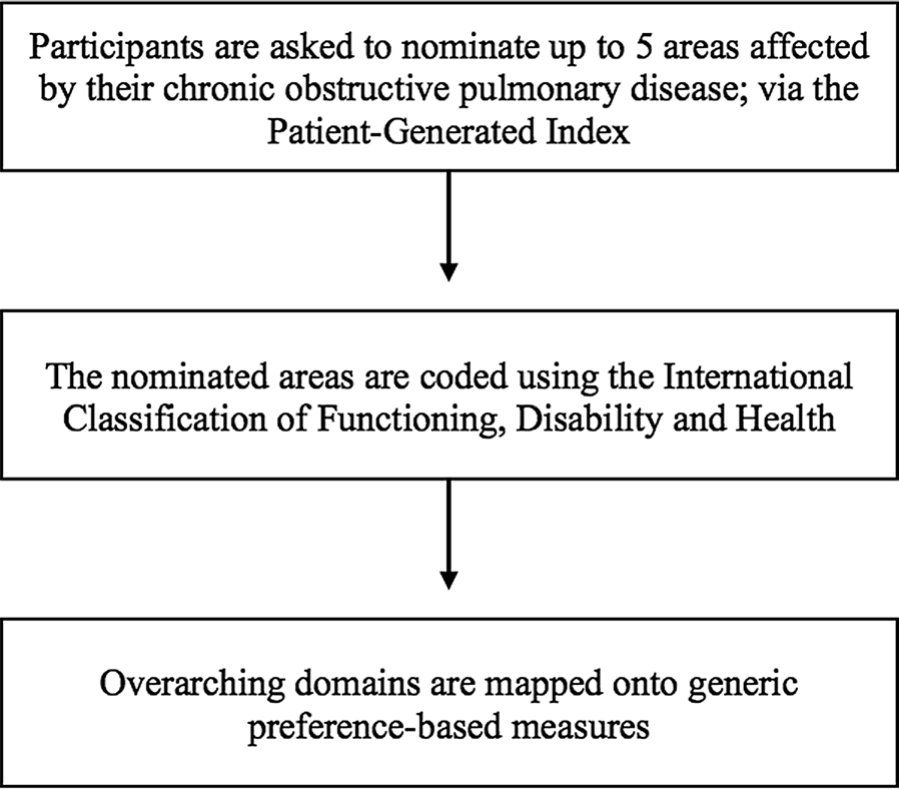
Flow diagram outlining the study’s procedure

Overarching domains were identified from the codes and mapped onto GPBMs: the EQ-5D, the SF-6D, the Health Utilities Index Mark 2 (HUI2), the Health Utilities Index Mark 3 (HUI3), the Assessment of Quality of Life 8-Dimensions (AQoL-8D), the 15-Dimensional (15D) and the Quality of Well-Being Self-Administered (QWB-SA) scale [3]. Mapping was also performed independently by two reviewers (AM and SO) with a third reviewer (AK) for consultation, if needed. This methodology followed previous studies examining content validity of GPBMs using the PGI [17, 18]. A flow diagram of the study’s procedure is outlined in Fig. 1.

### Statistical analysis

All statistical analyses were performed using Stata, version 15.1 (StataCorp, College Station, TX, USA). Descriptive statistics (mean and standard deviation, or frequency and percentage) were calculated to analyze participants’ sociodemographic/clinical information, ICF codes/domains identified and domains covered by GPBMs. A Pearson’s correlation coefficient was calculated to assess the correlation between the SF-6D and PGI scores. A positive correlation coefficient of at least 0.5 was hypothesized between the PGI and the SF-6D, as both measures are evaluating the construct of HRQoL [29].

### Sample size

There are no specific sample size estimates for content validation; therefore, our sample size was based on the number needed to achieve saturation. Common saturation guidelines agree that saturation for qualitative analysis is achieved at small sample sizes (e.g., around 20–30) and usually do not need to be greater than 60 [30].

## Results

### Sample characteristics

Table 1 outlines the clinical and sociodemographic characteristics for the study sample. For our 60 participants, the mean age of the sample was 70 years and approximately 57% were males. On average, participants had a smoking history of 44 pack-years; 45% used supplemental oxygen and 50% used a mobility aid (e.g., walker, cane, wheelchair). The mean FEV1% predicted of the sample was approximately 43, with the majority having severe to very severe airflow obstruction (GOLD stage 3–4) [6]. The most common comorbidities were cardiac and/or respiratory (e.g., asthma). The mean PGI score was approximately 35 out of 100, with 100 being the highest self-reported HRQoL. The mean SF-6D score was 0.57 out of 1, with 1 representing best HRQoL.

**Table 1:** Clinical and sociodemographic characteristics of sample (N = 60)

Characteristic	N (%) [unless specified otherwise]
Mean age (SD)	69.7 (7.99)
Males	34 (56.67)
Mean pack-years (SD)	43.71 (16.82)
Oxygen Use	27 (45.00)
Mobility Aid Use	30 (50.00)
Mean FEV1% predicted (SD)	42.98 (21.66)^a^
Mean FEV1/FVC % (SD)	45.84 (15.65)^b^
GOLD 1	3 (5.17)^a^
GOLD 2	17 (29.31)^a^
GOLD 3	18 (31.03)^a^
GOLD 4	20 (34.48)^a^
Cardiac comorbidities	41 (68.33)
Respiratory comorbidities	33 (55.00)
Rheumatology comorbidities	16 (26.67)
Gastro-intestinal comorbidities	16 (26.67)
Cancer comorbidities	13 (21.67)
Vascular comorbidities	11 (18.33)
Other co-morbidities	49 (81.67)
Mean PGI score (SD) [0–100]	34.55 (20.19)
Mean SF-6D score (SD) [0–1]	0.57 (0.09)

*FEV1* Forced expiratory volume in one second, *FVC* forced vital capacity, *N* sample size, *PGI* Patient-Generated Index, *SD* standard deviation

^a^Missing data (N = 58)

^b^Missing data (N = 54)

### Life areas important to COPD

Nineteen overarching domains were identified and thirteen appeared more than once. Table 2 presents the thirteen domains. The top three overarching domains were ‘mobility’ (25.93%), ‘recreation and leisure’ (25.19%) and ‘domestic life’ (19.26%). Specifically, ‘mobility’ included walking and using transportation, ‘recreation and leisure’ included socializing, hobbies and sports, and ‘domestic life’ included housework, preparing meals and shopping.

**Table 2:** Overarching domains identified more than once from the Patient-Generated Index (total n = 270)

Frequency n (%)	Overarching domain	ICF component	ICF codes	Code frequency n (%)
70 (25.93)	Mobility	Activities and participation	Walking	17 (6.30)
			Mobility	11 (4.07)
			Using transportation	10 (3.7)
			Walking long distances	8 (2.96)
			Climbing	6 (2.22)
			Swimming	5 (1.85)
			Moving around outside the home and other buildings	5 (1.85)
			Walking on different surfaces	3 (1.11)
			Running	2 (0.74)
			Driving motorized vehicles	2 (0.74)
			Driving human-powered transportation	1 (0.37)
68 (25.19)	Recreation and leisure	Activities and participation	Socializing	22 (8.15)
			Hobbies	17 (6.30)
			Sports	12 (4.44)
			Play	8 (2.96)
			Recreation and leisure	5 (1.85)
			Community, social and civic life, other specified	3 (1.11)
			Arts and culture	1 (0.37)
52 (19.26)	Domestic life	Activities and participation	Housework	19 (7.04)
			Preparing meals	9 (3.33)
			Cleaning living area	7 (2.59)
			Shopping	5 (1.85)
			Taking care of plants, indoors and outdoors	3 (1.11)
			Maintaining dwelling and furnishings	2 (0.74)
			Washing and drying clothes and garments	2 (0.74)
			Domestic life	2 (0.74)
			Taking care of animals	1 (0.37)
			Caring for household objects	1 (0.37)
			Maintaining domestic appliances	1 (0.37)
28 (10.37)	Interpersonal relationships	Activities and participation	Family relationships	13 (4.81)
			Informal relationships with friends	5 (1.85)
			Sexual relationships	4 (1.48)
			Interpersonal interactions and relationships	3 (1.11)
			Informal social relationships	2 (0.74)
			Parent–child relationships	1 (0.37)
10 (3.7)	Mental functions	Activities and participation	Emotional functions	6 (2.22)
			Energy level	2 (0.74)
			Openness to experience	1 (0.37)
			Confidence	1 (0.37)
8 (2.96)	Work and employment	Activities and participation	Remunerative employment	7 (2.59)
			Non-remunerative employment	1 (0.37)
6 (2.22)	Carrying/lifting objects	Activities and participation	Lifting and carrying	3 (1.11)
			Lifting	2 (0.74)
			Carrying in the hands	1 (0.37)
5 (1.85)	Self-care	Activities and participation	Washing whole body	5 (1.85)
4 (1.48)	Changing/maintaining body position	Activities and participation	Maintaining a standing position	2 (0.74)
			Bending	1 (0.37)
			Standing	1 (0.37)
4 (1.48)	Environmental factors	Environmental factors	Climate	4 (1.48)
4 (1.48)	Carrying out daily routine	Activities and participation	Carrying out daily routine	3 (1.11)
			Managing one’s own activity level	1 (0.37)
3 (1.11)	Respiratory system functions	Body functions	Respiratory functions	3 (1.11)
2 (0.74)	Looking after one’s health	Activities and participation	Maintaining one’s health	2 (0.74)

*ICF* World Health Organization’s International Classification of Functioning, Disability and Health, *n* number of appearances

**Fig. 2:**
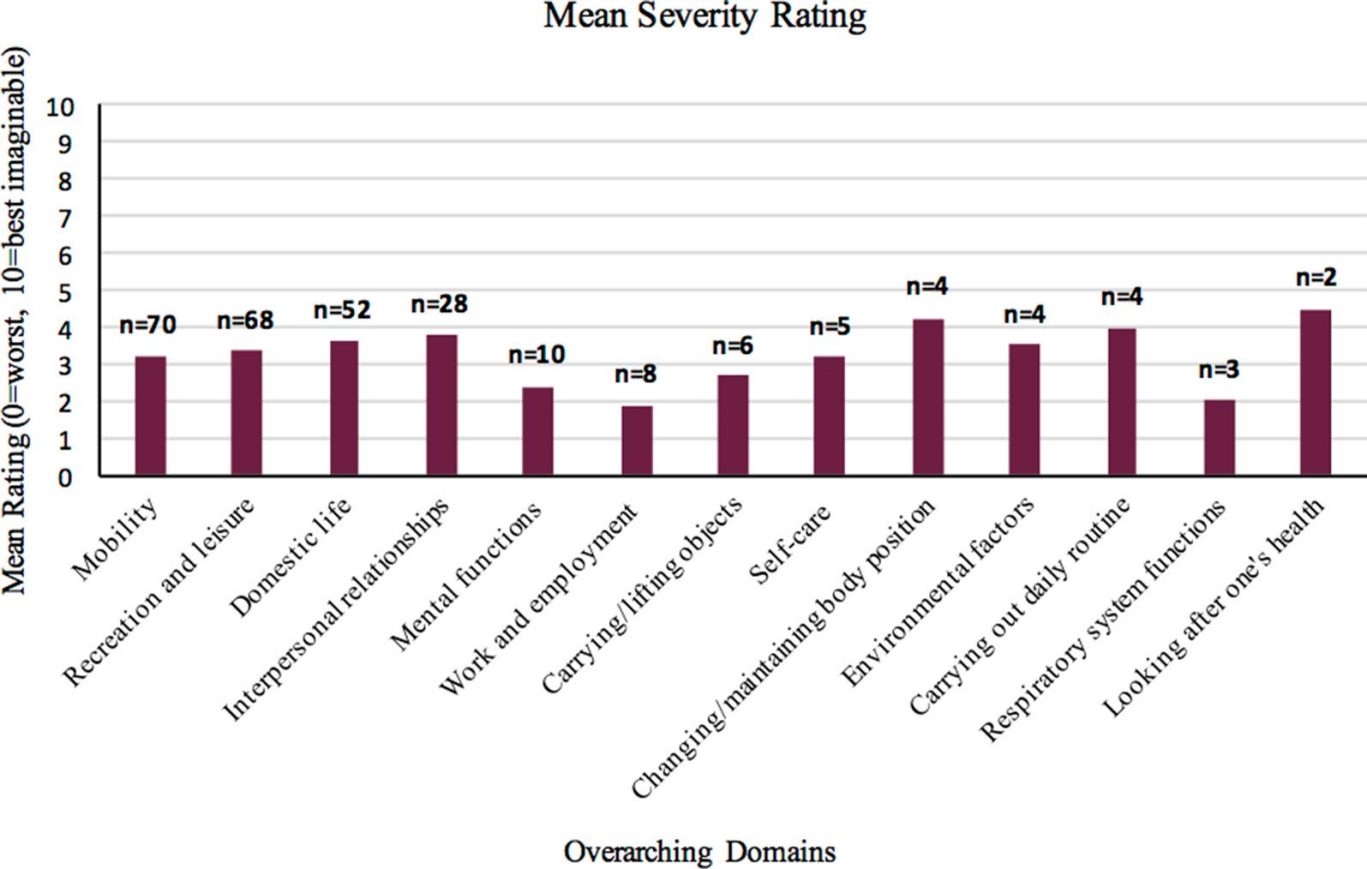
Mean severity rating given to each overarching domain appearing more than once, scaled from 0 (the worst one could imagine) to 10 (exactly as one would like it to be) n = number of appearances

Figure 2 outlines the mean severity rating (from 0 to 10, where 0 is the worst and 10 is the best one could imagine that area to be) of each overarching domain. Although, ‘work and employment’ was reported only 8 times, it was found to be the area most severely impacted by COPD with a mean score close to 2 out of 10 (very poor). ‘Mobility’, ‘recreation and leisure’, ‘domestic life’ and ‘interpersonal relationships’ were also severely affected with mean scores ranging from 3 (poor) to 4 (between poor and fair).

**Fig. 3:**
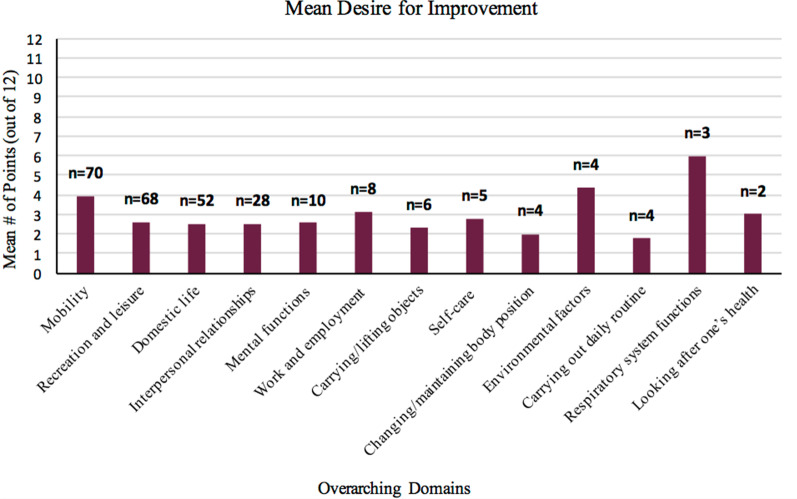
Mean number of points (out of 12) for improvement desires allocated to each overarching domain appearing more than once n = number of appearances

Figure 3 outlines the mean number of points (out of 12) that participants allocated to the overarching domains, indicating their desire for improvement in that area. With a frequency of 3, ‘respiratory system functions’ (e.g., breathing) was the area most desired for improvement (mean 6 points; 50% of their points), followed by ‘environmental factors’ (e.g., weather conditions) (mean 4.4 points; 37% of their points) and ‘mobility’ (mean 4 points; 33% of their points). Participants’ spent on average 2.5 points (21% of their points) on ‘recreation and leisure’, ‘domestic life’, ‘interpersonal relationships’ and ‘mental functions’ each.

### Content validity

Table 3 presents the mapping of the overarching domains against items on the GPBMs. The QWB-SA covered the highest number of domains important to individuals with COPD (84.62%) and the HUIs covered the least (15.38% and 30.77%). The rest of the GPBMs covered between 46 and 62%. ‘Mobility’ and ‘mental functions’ domains were covered by all the measures, and ‘environmental factors’ and ‘looking after one’s health’ were not covered by any of the measures. ‘Recreation and leisure’ and ‘domestic life’, areas commonly reported by participants, were covered by the EQ-5D, SF-6D, AQoL-8D, 15D and QWB-SA, but not by HUI2 and HUI3. ‘Interpersonal relationships’ was covered by the AQoL-8D, 15D and QWB-SA, but not by EQ-5D, SF-6D, HUI2 and HUI3.

**Table 3:** Mapping of overarching domains, identified by COPD patients, onto GPBMs

Overarching domains	Generic preference-based measure						
	EQ-5D	SF-6D	HUI2	HUI3	AQoL-8D	15D	QWB-SA
Mobility	Y	Y	Y	Y	Y	Y	Y
Recreation and leisure	Y	Y	N	N	Y	Y	Y
Domestic life	Y	Y	N	N	Y	Y	Y
Interpersonal relationships	N	N	N	N	Y	Y	Y
Mental functions	Y	Y	Y	Y	Y	Y	Y
Work and employment	Y	Y	N	N	N	Y	Y
Carrying/lifting objects	N	N	Y	N	N	N	Y
Self-care	Y	Y	Y	N	Y	N	Y
Changing/maintaining body position	N	N	N	N	N	N	Y
Environmental factors	N	N	N	N	N	N	N
Carrying out daily routine	Y	Y	N	N	N	Y	Y
Respiratory system functions	N	N	N	N	N	Y	Y
Looking after one’s health	N	N	N	N	N	N	N
% of Yes	53.85%	53.85%	30.77%	15.38%	46.15%	61.54%	84.62%

*Y* yes, it is covered by the measure, *N* no, it is not covered by the measure, *EQ-5D* EuroQol 5-Dimensions, *SF-6D* Six-Dimensional Short Form Survey, *HUI 2* Health Utilities Index Mark 2, *HUI 3* Health Utilities Index Mark 3, *AQoL-8D* Assessment of Quality of Life 8-Dimensions, *15D* 15-Dimensional, *QWB-SA* Quality of Well-Being Self-Administered

### Convergent validity

**Fig. 4:**
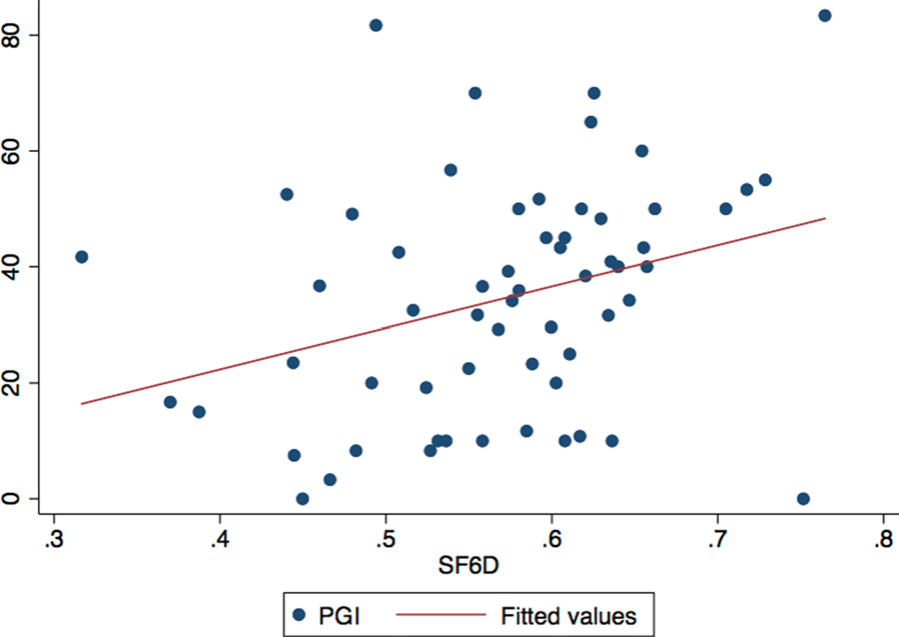
Scatter plot of SF-6D scores against Patient-Generated Index scores with a line of best fit

A Pearson’s correlation coefficient of 0.32 was calculated between the PGI and the SF-6D. Figure 4 presents a scatter plot of SF-6D scores against PGI scores. Correlation values between the two measures did not fall around the line of best fit and were scattered, but did follow an upward trend, indicating a weak positive correlation between the measures [31].

## Discussion

This was the first study to evaluate the content validity of GPBMs in individuals with COPD [8]. Areas of life most affected by COPD were identified by people with COPD, coded using the ICF and mapped onto GPBMs. A major finding of this study was that the majority of GPBMs covered only half of the areas reported as being important to individuals with COPD. In particular, several domains, such as respiratory problems, interpersonal relationships and work and employment, were missing from one or more of the GPBMs. We also found the SF-6D, a well-known GPBM, to be weakly associated with the PGI, an individualized measure of HRQoL capturing issues COPD patients consider important. Taken together, these findings suggest that GPBMs may not necessarily be suitable for assessing the HRQoL of COPD patients for cost-effectiveness analyses.

Many of the domains reported by patients with COPD were both severely affected and had a large proportion of points allocated to them, indicating their importance to participants. Mobility, for example, was not only an area that was severely impacted, but also an area that participants desired to improve notably. Without mobility, other aspects of life may become impaired. Being able to leave one’s house can help expand one’s social circle and allow for engagement in meaningful activities [32]. Similarly, physical movement is needed to engage in sports or perform chores around the house. This was evident in our findings as individuals with COPD highly reported social and participation restrictions in addition to mobility. Respiratory function was the second most impacted area by COPD and was given the highest amount of points in terms of desire for improvement. Even though this area was not highly reported, this finding suggests that among those listing it as important, they found it to be severely impacted by COPD and valued it highly by allocating, on average, half of their points to this area.

One of the biggest advantages of GPBMs is that they can be used for economic evaluation purposes to determine the cost-utility of alternative treatments and programs. They allow the different dimensions of health to be combined into a single index with anchors from 0 (death) to 1 (perfect health). GPBMs attach explicit weights to the various dimensions of health, allowing trade-offs to be made between them [24]. However, in the context of COPD, the majority of GPBMs, including the most widely used GPBM for cost-effectiveness analysis; the EQ-5D [3], only covered approximately half of the areas reported as being important to patients. Interpersonal relationships, a frequently reported affected area, along with carrying/lifting objects, changing/maintaining body positions and respiratory problems were not covered by the majority of these measures. If such aspects are not captured by preference-based measures, then the overall HRQoL score may be inaccurate in terms of its reflection of patients’ values, and thus, the cost-effectiveness of healthcare interventions and decisions made based on these results may also be inaccurate.

The HUIs covered less than one third of the areas nominated by COPD patients. The HUI3 evolved from the HUI1 and HUI2 [33], which were originally developed for infants and children [34, 35]. Although the HUI2 has been applied in older populations (i.e., Alzheimer’s disease) [36], its validity was not tested and some domains, such as ‘fertility’, remain relevant to younger populations. HUI2 and HUI3 focus on sensory difficulties, which is not necessarily relevant to a respiratory disease population. The HUIs were developed using the “within the skin” definition of health status, which focuses on impairments and excludes social interactions [33, 37]. Therefore, frequently reported areas, such as recreation and leisure, domestic life and interpersonal relationships, that encompass social aspects of HRQoL were not covered by these measures.

The QWB-SA is a comprehensive measure of HRQoL encompassing 58 symptoms (mental, acute physical and chronic) [38]. Even though the QWB-SA covered many of the life areas reported by participants, it is not as widely used as other preference-based measures like the EQ-5D [3]. This may be because it consists of 71 items and has a 14-min completion time in older adults [39], compared to the EQ-5D which consists of 5 items and only takes a few minutes to complete [40]. Furthermore, the QWB-SA is heavily focused on symptoms, which can be burdensome for respondents if they do not possess the listed symptoms. In our study, when asked about the important areas of life affected by COPD, none of the chronic symptoms and only 2 of the acute symptoms on the QWB-SA were mentioned by participants (i.e., shortness of breath and difficulty walking/standing). Having HRQoL measures with short administration times that target important areas affected by COPD may be valuable, providing accurate and easy to implement tools for cost-effectiveness analyses in clinical trials focused on patients with COPD.

A limitation of this study is that the sample comprised a low percentage of individuals with mild airflow limitations (5.17%). A recent study using data from the Canadian Cohort Obstructive Lung Disease (CanCOLD) study found two-thirds of the cohort to be undiagnosed for COPD [41]. These individuals were not given a clinical diagnosis but had airflow obstruction according to spirometry tests [41]. Even though individuals with mild airflow limitations present fewer symptoms [42], they compose a large portion of the population and their perspectives may have not been completely captured in our study. However, the disease severity of our sample was comparable to other COPD samples in the measurement literature [43–47]. A second limitation of this study is the comparability of findings to other healthcare settings. Since recruitment was performed at tertiary care settings, findings may not be transferable to other settings (e.g., primary care settings). Last, for the PGI, participants were asked to list the most important areas of their life affected by their COPD. The phrasing of this question elicits reference to life activities and may result in less identification of the symptoms relevant to the disease. For example, respiratory system functions such as difficulty breathing, well-known to impact the COPD population [6], were not highly endorsed by this sample.

## Conclusions

GPBMs form the basis for cost-effectiveness analysis and resource allocation decisions within the healthcare system, however, our findings showed that not a single measure covered all life areas important to those living with COPD and that their association with an individualized measure of HRQoL is weak. The content of preference-based measures should be reflective of the population’s health concerns for accurate economic evaluation of treatments [48]. When GPBMs are used to evaluate the cost-utility of interventions in COPD, they may not always be sensitive to the concerns and values of individuals with COPD, which may result in inaccurate recommendations. Findings from this study suggest that a COPD-specific preference-based measure could be developed in order to more accurately reflect the health concerns of individuals living with COPD. Until such a measure is developed, researchers and policymakers can use these findings to make informed decisions when selecting a GPBM for cost-effectiveness analyses of interventions in the COPD population.

## Data Availability

The datasets generated and/or analysed during the current study are not publicly available due to privacy and confidentiality reasons but are available from the corresponding author on reasonable request.

## Abbreviations

15D: 15-Dimensional
AQoL-8D: Assessment of Quality of Life 8-Dimensions
COPD: Chronic Obstructive Pulmonary Disease
EQ-5D: EuroQol 5-Dimensions
FEV1: Forced Expiratory Volume in 1 s
FVC: Forced Vital Capacity
GPBM: Generic Preference-Based Measure
HRQoL: Health-Related Quality of Life
HUI 2: Health Utilities Index Mark 2
HUI 3: Health Utilities Index Mark 3
ICF: International Classification of Functioning, Disability and Health
PGI: Patient-Generated Index
QALYs: Quality-Adjusted Life Years
QWB-SA: Quality of Well-Being Self-Administered
SF-6D: Six-Dimensional Short Form Survey
